# *Porphyromonas gingivalis* disrupts vascular endothelial homeostasis in a TLR-NF-κB axis dependent manner

**DOI:** 10.1038/s41368-020-00096-z

**Published:** 2020-09-30

**Authors:** Mengru Xie, Qingming Tang, Shaoling Yu, Jiwei Sun, Feng Mei, Jiajia Zhao, Lili Chen

**Affiliations:** 1 Department of Stomatology, Union Hospital, Tongji Medical College, Huazhong University of Science and Technology, Wuhan, China; 2 Hubei Province Key Laboratory of Oral and Maxillofacial Development and Regeneration, Wuhan, China

**Keywords:** Bacterial pathogenesis, Mechanisms of disease

## Abstract

Cardiovascular disease is still the leading cause of mortality worldwide. Vascular endothelial dysfunction is viewed as the initial step of most cardiovascular diseases. Many studies have indicated that periodontal pathogens, especially *Porphyromonas gingivalis*, are closely correlated with vascular endothelial homeostasis, but the function of *P. gingivalis* and the underlying mechanisms are still elusive. To illuminate the effects and elucidate the mechanisms of *P. gingivalis* on endothelial structural integrity, we developed *P. gingivalis* infection models in vivo and in vitro. Endothelial cell proliferation, differentiation and apoptosis were detected. Here, we showed that *P. gingivalis* can impair endothelial integrity by inhibiting cell proliferation and inducing endothelial mesenchymal transformation and apoptosis of endothelial cells, which reduce the cell levels and cause the endothelium to lose its ability to repair itself. A mechanistic analysis showed that TLR antagonist or NF-κB signalling inhibitor can largely rescue the damaged integrity of the endothelium caused by *P. gingivalis*, suggesting that TLR-NF-κB signalling plays a vital role in vascular endothelial homeostasis destroyed by *P. gingivalis*. These results suggest a potential intervention method for the prevention and treatment of cardiovascular disease.

## Introduction

Despite significant advances in the clinical treatment of cardiovascular disease, this condition is still the leading cause of death worldwide, highlighting an important yet unmet clinical need.^1^ The mechanisms involved in cardiovascular disease are complex. Recently, an increasing number of researchers have regarded inflammatory processes as central to the development and complications of cardiovascular disease.^2–4^ Accumulating evidence has shown that infections with certain microorganisms, such as *Chlamydia pneumoniae*,^5,6^ human immunodeficiency virus (HIV)^7^ and others,^8–10^ may be implicated in these processes. Recently, periodontal pathogens have attracted attention. These pathogens can gain access to the systemic circulation during common activities, such as brushing, flossing, and chewing, as well as during dental procedures,^11^ so they not only cause local inflammation and tissue destruction but also have systemic effects on organisms. Periodontal diseases caused by these pathogens show an epidemiological correlation with cardiovascular disease.^12–14^ Some researchers have already observed the existence of periodontal pathogens in lesions of the cardiovascular system.^15–17^ Moreover, in vivo studies have demonstrated an adverse association between periodontal pathogens and cardiovascular disease.^18–20^ In addition, periodontal therapy showed a reduction in systemic and aortic inflammation caused by periodontitis.^21^ These reports support the hypothesis that periodontal pathogen infection accounts for the pathogenesis of cardiovascular disease.

*Porphyromonas gingivalis*, a Gram-negative anaerobic bacterium, is a major periodontal pathogen. A recent study found that all patients with cardiovascular disease had *P. gingivalis* arterial colonization,^15^ suggesting an obvious link between *P. gingivalis* and cardiovascular disease. Animal studies have reported that oral infection with *P. gingivalis* accelerates atherosclerosis,^19^ and inoculation with *P. gingivalis* caused a significant increase in mortality due to cardiac rupture.^22^ It is known that *P. gingivalis* can frequently enter the vasculature. There are two possible ways. The gingival area of the periodontal region is surrounded by a multitude of lymph vessels with an open junction that can capture and trap bacteria.^23^ *P. gingivalis* can directly enter the vasculature via these lymph vessels. Lymph vessels can return extravasated fluid, proteins, and cells back into the circulation.^24^ In addition, this bacterium can enter the vasculature by trafficking by inflammatory cells from the local site of infection (such as the oral cavity). In this model, as a result of the tissue damage caused by the infection and subsequent inflammatory response, the inflammatory cells present at the local infection ingest the pathogens, and these pathogen-laden inflammatory cells move into the vasculature.^25^ In the vasculature, *P. gingivalis* can adhere to and invade vascular endothelial cells, whose normal functioning is crucial for maintaining vascular health and whose dysfunction is key in the initiation, progression and clinical complications of vascular disease.^26^ Endothelial cells stimulated with *P. gingivalis* produce high levels of proinflammatory cytokines, cell adhesion molecules, chemokines and nitric oxide,^27,28^ which have important effects on vascular function. These results indicate the association between *P. gingivalis* and endothelial function, strongly suggesting that *P. gingivalis*-induced endothelial dysfunction may be involved in the progression of cardiovascular disease. Nevertheless, it is compulsory to investigate the effects of *P. gingivalis* on endothelial biological properties and to elucidate the contribution of possible activation of signalling pathways.

Our results showed that *P. gingivalis* can inhibit the proliferation and induce endothelial mesenchymal transformation (EndMT) and apoptosis of endothelial cells by activating TLR-NF-κB signalling. *P. gingivalis* destroys vascular endothelial homeostasis by reducing its income and increasing expenditure, which provides a potential intervention method for the prevention and treatment of cardiovascular disease.

## Results

### *P. gingivalis* can severely damage vascular endothelial integrity

**Fig. 1:**
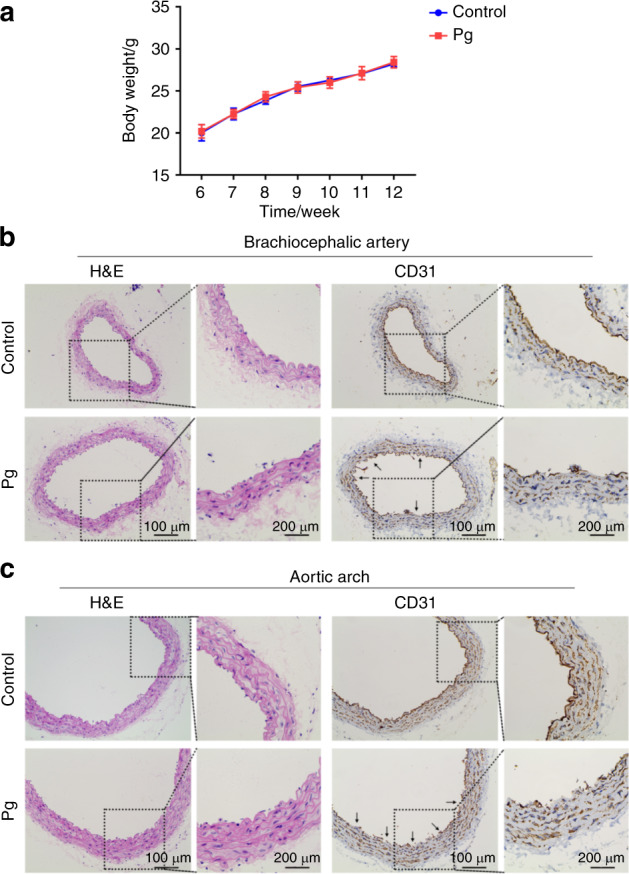
*P. gingivalis* infection damages vascular endothelial integrity. **a** The weight of mice in the control and *P. gingivalis* (Pg) groups at 6, 7, 8, 9, 10, 11, and 12 weeks. Representative images after haematoxylin-eosin staining (H&E) and CD31 immunohistochemistry (IHC) of brachiocephalic artery (**b**) and aortic arch (**c**) tissue from the Balb/c mice with Pg tail-vein injection for 6 weeks (*n* = 5). Black arrow, the site where endothelial integrity was destroyed. Data were analysed using two-tailed unpaired *t*-tests (**a**) and are presented as the mean ± SD

To examine the effects of *P. gingivalis* on vascular endothelial integrity, we used Balb/c mice as a model by tail-vein injection of *P. gingivalis* (10^7^ CFU per mouse) once a week for 6 weeks. First, we measured the weight of the mice, and we found that there was no significant difference between the control and Pg (*P. gingivalis*) groups (Fig. 1a). Then, the mouse aorta was separated and stained with haematoxylin-eosin (H&E) for morphological detection and for the endothelial marker cluster of differentiation 31 (CD31) for endothelial integrity observation. In the control mice, the endothelium of the brachiocephalic artery (Fig. 1b) and aortic arch (Fig. 1c) were both intact. In the *P. gingivalis*-infected mice, the continuity of the endothelium was broken, showing that the anatomical integrity of the endothelium was damaged by *P. gingivalis*.

### *P. gingivalis* can obviously inhibit the proliferation of endothelial cells

**Fig. 2:**
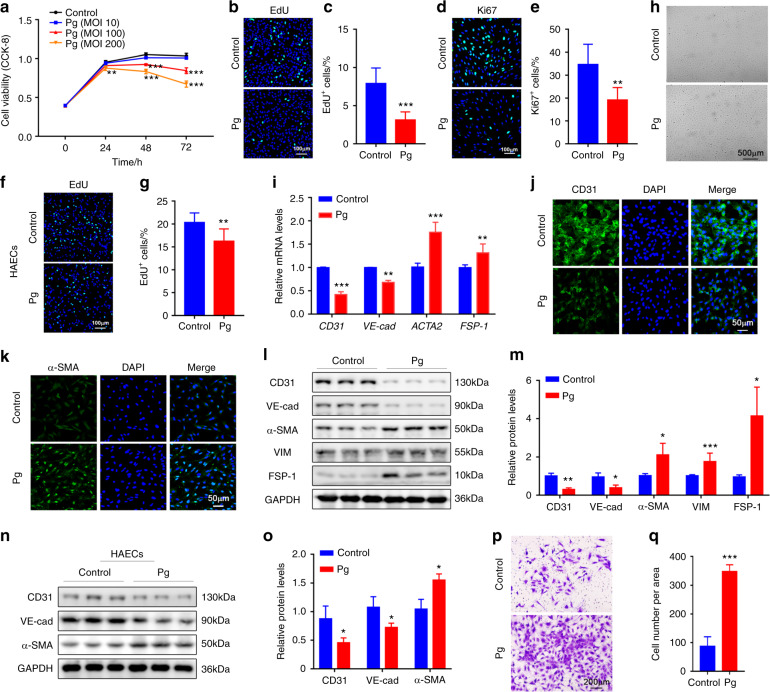
*P. gingivalis* inhibits proliferation and stimulates endothelial mesenchymal transformation (EndMT) in cultured endothelial cells. **a** The viability of human umbilical vein endothelial cells (HUVECs) treated with *P. gingivalis* (Pg) at different concentrations was measured by CCK-8 assays. MOI, multiplicity of infection. **b** EdU staining of HUVECs after Pg exposure for 48 h. Nuclei were stained with DAPI (blue). **c** The quantitative data of (**b**). **d** Immunofluorescence (IF) staining of Ki67 (green) in HUVECs with or without Pg infection for 48 h. **e** The quantitative data of (**d**). **f** EdU staining of human aortic endothelial cells (HAECs) after Pg exposure for 48 h. Nuclei were stained with DAPI (blue). **g** The quantitative data of (**f**). **h** HUVECs showed remarkable morphological changes after Pg treatment for 48 h. Representative images are presented. **i** Relative CD31, VE-cadherin (VE-cad), ACTA2 and FSP-1 mRNA levels were measured by real-time polymerase chain reaction (qRT-PCR) in the control or Pg-treated HUVECs. IF staining of CD31 (**j**) and α-SMA (**k**) in HUVECs after Pg infection or not. **l** Western blot analysis of the EndMT-related protein levels in the HUVECs after Pg exposure. **m** The quantitative data of **l**. **n** Western blot analysis of EndMT-related protein levels in HAECs after Pg exposure. **o** The quantitative data of (**n**). **p** Transwell assays were used to test the migration of the control and Pg-infected HUVECs. **q** The quantitative data of (**p**). Data were analysed using two-tailed unpaired *t*-tests (**c**, **e**, **g**, **i**, **m**, **o**, **q**) and are presented as the mean ± SD. *P < 0.05; **P < 0.01; ***P < 0.001 compared to the control group

The anatomical integrity of the endothelium is maintained by the balance of the sources and outlets of endothelial cells. The proliferation of endothelial cells is a key source of endothelium and is viewed as very important in the repair of areas denuded of endothelium as a result of injury.^26^ We first measured the proliferative ability of endothelial cells in vitro. Human umbilical endothelial cells (HUVECs) were treated with *P. gingivalis* at different multiplicities of infection (MOIs) for various periods of time. We observed a significant decrease in the viability of HUVECs at both higher MOIs (100 and 200) in the 48-h and 72-h groups (Fig. 2a). A low MOI (10) did not show these results (Fig. 2a). EdU assays and Ki67 staining were used to further detect the proliferation of HUVECs. The data showed that the number of proliferating cells was decreased significantly after *P. gingivalis* infection (MOI 100) for 48 h (Fig. 2b–e). In addition to HUVECs, we observed reduced proliferation of human aortic endothelial cells (HAECs) after *P. gingivalis* exposure (Fig. 2f, g). These data suggested that the decreased cell proliferation of endothelial cells is likely to be closely related to vascular endothelial injury.

### *P. gingivalis* induces endothelial mesenchymal transformation of endothelial cells

Endothelial mesenchymal transformation (EndMT) is a crucial step in endothelial cell differentiation to several lineages, and it has been demonstrated to be involved in many cardiovascular diseases.^29^ Due to EndMT, the endothelium can lose its integrity as a barrier in blood vessels.^30^ To test whether *P. gingivalis* could affect the EndMT process, we treated HUVECs with *P. gingivalis* for 48–72 h. *P. gingivalis* treatment led to striking morphological changes in HUVECs (Fig. 2h). The phenotype of endothelial cells was altered from a typical cobblestone-like to a fibroblast-like appearance, and the number of elongated and spindle-like HUVECs increased significantly (Fig. 2h). In accordance with the morphological change, the expression of the endothelial-specific markers CD31 and vascular endothelial cadherin (VE-cadherin) were reduced, and the mesenchymal markers fibroblast-specific protein (FSP-1) and α-smooth muscle actin (α-SMA) were increased in the *P. gingivalis*-treated HUVECs compared to the controls, both at the mRNA and protein levels (Fig. 2i–m). The relative protein level of vimentin (VIM), another EndMT marker, was also increased significantly (Fig. 2l, m). A similar change was also observed in HAECs (Fig. 2n, o). Except for the molecular changes, one of the characteristics of EndMT is that cells lose cell-cell adhesion and acquire an enhanced migratory phenotype. We performed a cell transwell assay to measure the migration of HUVECs. After incubation with *P. gingivalis* for 48 h, the number of migrated HUVECs was increased significantly, approximately 3.4-fold compared to that of the controls (Fig. 2p, q). Together, our findings indicated that *P. gingivalis* infection induced the differentiation of endothelial cells.

### *P. gingivalis* triggers the apoptosis of endothelial cells

**Fig. 3:**
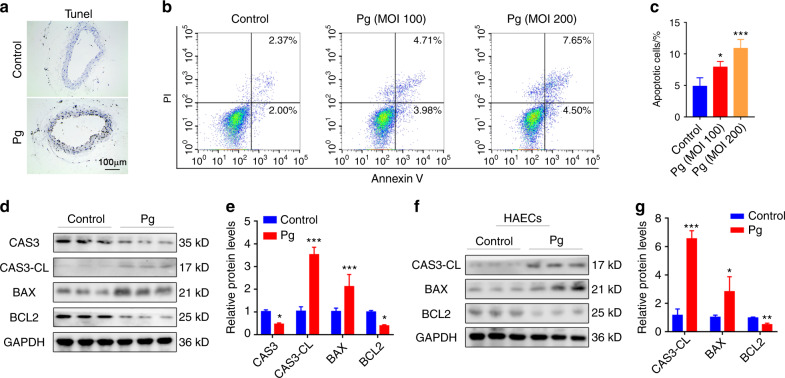
*P. gingivalis* triggers endothelial apoptosis. **a** Apoptosis in vivo was assessed by TUNEL staining in brachiocephalic artery tissue from the Balb/c mice treated with or without *P. gingivalis* (Pg) (*n* = 5). **b** Apoptosis was evaluated by flow cytometry of human umbilical vein endothelial cells (HUVECs) treated with Pg for 24 h. **c** The quantitative data of (**b**). **d** Western blot analysis of the indicated protein levels in HUVECs after Pg stimulation for 24 h. **e** The quantitative data of (**d**). **f** Western blot analysis of the indicated protein levels in human aortic endothelial cells (HAECs) after Pg stimulation for 24 h. **g** The quantitative data of (**f**). Data were analysed using a one-way ANOVA (**c**) or two-tailed unpaired *t*-tests (**e**, **g**) and are presented as the mean ± SD. *P < 0.05; **P < 0.01; ***P < 0.001 compared to the control group

Endothelial cell apoptosis induced by risk factors is believed to lead to the denudation or dysfunction of the intact endothelial monolayer.^31^ To explore the reason for the loss of endothelial integrity, we next measured the apoptosis of endothelial cells. TUNEL staining of the brachiocephalic artery showed that after *P. gingivalis* infection, the number of apoptotic endothelial cells was increased (Fig. 3a). We then treated HUVECs with *P. gingivalis* at different concentrations for 24 h and found that the ratio of apoptotic cells was obviously elevated after *P. gingivalis* infection, from ~5% (control) to ~7.5% (MOI 100) and ~11% (MOI 200) (Fig. 3b, c). The expression of the proapoptotic proteins cleaved Caspase 3 (CAS-CL) and BAX was increased, and the expression of the antiapoptotic proteins Caspase 3 and BCL2 was decreased not only in the *P. gingivalis*-infected HUVECs (Fig. 3d, e) but also in the *P. gingivalis*-infected HAECs (Fig. 3f, g). Collectively, these observations demonstrated that *P. gingivalis*-destroyed vascular endothelial homeostasis mainly by inhibiting the proliferation and inducing EndMT and apoptosis of endothelial cells.

### P. gingivalis destroys vascular endothelial homeostasis by combining with membranous TLRs

**Fig. 4:**
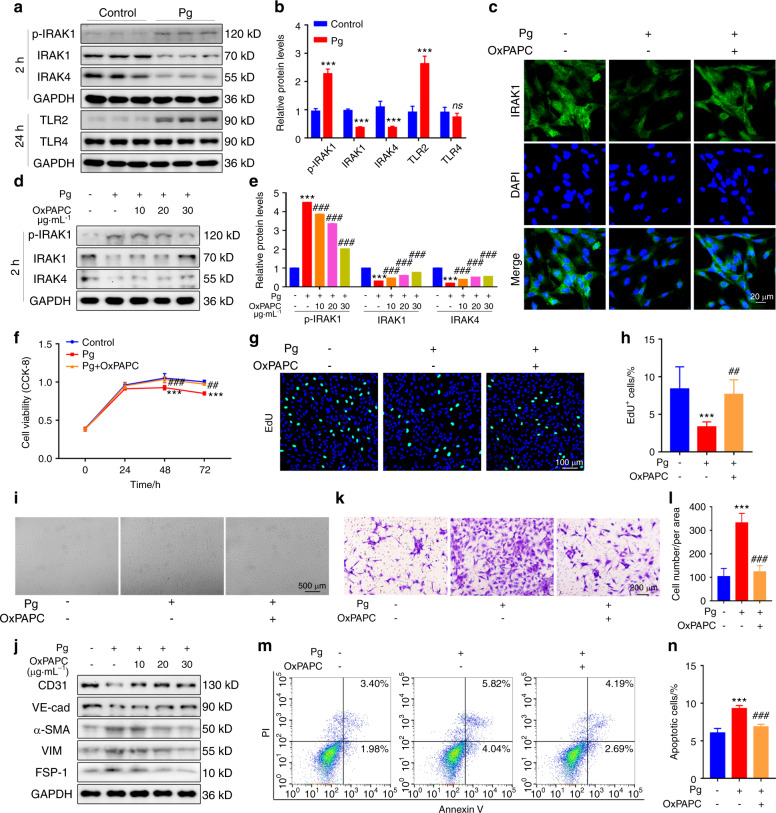
*P. gingivalis* damages the integrity of endothelial cells through TLRs. **a** Western blot analysis of TLR signalling-related protein levels in human umbilical vein endothelial cells (HUVECs) after *P. gingivalis* (Pg) exposure for 2 h or 24 h. **b** The quantitative data of (**a**). **c** Confocal images of IRAK1 expression in HUVECs treated with Pg and/or the TLR antagonist OxPAPC (30 μg·mL^−1^) for 2 h. **d** Western blot analysis of the p-IRAK1, IRAK1, and IRAK4 protein levels in the HUVECs treated with Pg and/or OxPAPC (10, 20, 30 μg·mL^−1^) for 2 h. **e** The quantitative data of (**d**). **f** The viability of HUVECs treated with Pg and/or OxPAPC at different timepoints was measured by CCK-8 assays. **g** EdU staining of HUVECs treated with Pg and/or OxPAPC (30 μg·mL^−1^) for 48 h. Nuclei were stained with DAPI (blue). **h** The quantitative data of (**g**). **i** HUVECs showed remarkable morphological changes after Pg and/or OxPAPC treatment for 48 h. Representative images are presented. **j** Western blot analysis of the EndMT-related protein levels in the HUVECs treated with Pg and/or OxPAPC (10, 20, 30 μg·mL^−1^) for 72 h. **k** Transwell assays were used to test the migration of HUVECs treated with Pg and/or OxPAPC (30 μg·mL^−1^) for 48 h. **l** The quantitative data of (**k**). **m** Apoptosis was evaluated by flow cytometry of the HUVECs treated with Pg and/or OxPAPC (30 μg·mL^−1^) for 24 h. **n** The quantitative data of (**m**). Data were analysed using a two-tailed unpaired *t*-test (**b**) or one-way ANOVA (**e**, **h**, **l**, **n**) or a two-way ANOVA (**f**) and are presented as the mean ± SD. *P < 0.05; **P < 0.01; ***P < 0.001 compared to the control group. ns = not significant. ^#^*P* < 0.05; ^##^*P* < 0.01; ^###^*P* < 0.001 compared to the Pg group

Toll-like receptors (TLRs) are thought to mediate *P. gingivalis* recognition and in turn activate downstream signalling pathways to regulate host cell responses.^32^ Similarly, we detected activated TLR downstream signalling in HUVECs after *P. gingivalis* infection (Fig. 4a, b). To further evaluate whether TLRs participated in *P. gingivalis*-induced vascular endothelial damage, the TLR signalling antagonist OxPAPC was used. OxPAPC inhibited *P. gingivalis*-activated TLR signalling in a dose-dependent manner (Fig. 4c–e), and the effects of *P. gingivalis* on proliferation, EndMT and apoptosis of HUVECs were also reversed (Fig. 4f–n). The CCK-8 assay showed that the viability of HUVECs was almost completely recovered when OxPAPC was added (Fig. 4f). Consistently, the ratio of EdU-positive cells was increased to the comparable level of control cells when OxPAPC was used (Fig. 4g, h). Next, we examined the change in morphology, the expression of EndMT markers and the migration of HUVECs with OxPAPC treatment. As shown, *P. gingivalis* led to striking morphological changes in HUVECs, but these changes were attenuated by OxPAPC (Fig. 4i). Additionally, the downregulated expression of CD31 and VE-cadherin and the upregulated expression of α-SMA, VIM and FSP-1 were reversed by OxPAPC (Fig. 4j). In addition, the transwell assay showed that the enhanced migration of *P. gingivalis*-treated HUVECs was alleviated almost to the same level of control cells by OxPAPC treatment (Fig. 4k, l), indicating that blockade of the TLR signalling pathway reversed the process of EndMT. In addition, we detected a decrease in apoptosis in the OxPAPC-treated HUVECs. The ratio of apoptotic cells was elevated after *P. gingivalis* infection, and it was reduced after treatment with OxPAPC (Fig. 4m, n). Together, our results revealed that TLRs account for the *P. gingivalis*-mediated impairment of the integrity of the endothelium.

### NF-κB signalling plays a critical role in *P. gingivalis*-destroyed endothelial homeostasis

**Fig. 5:**
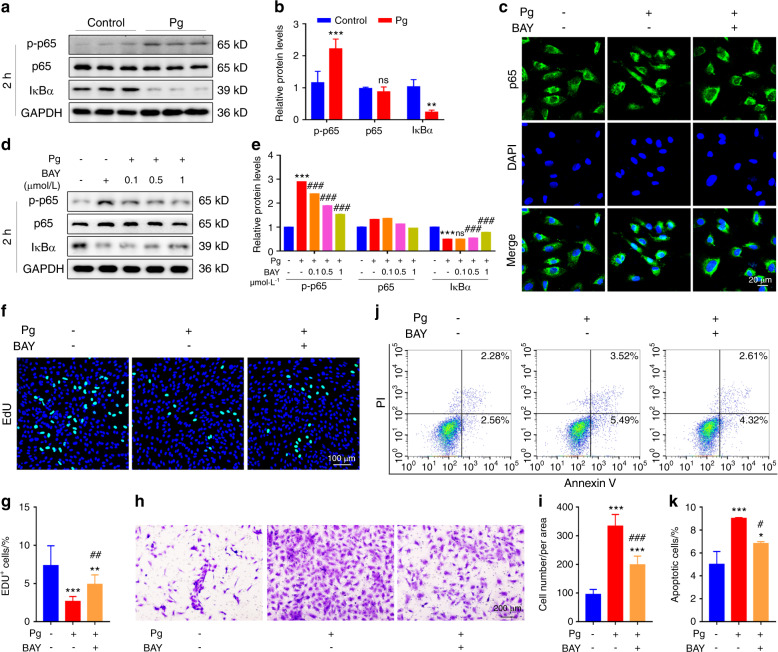
NF-kappaB signalling plays a critical role in *P. gingivalis*-destroyed endothelial homeostasis. **a** Western blot analysis of NF-kappaB (NF-κB) signalling-related protein levels in human umbilical vein endothelial cells (HUVECs) after *P. gingivalis* (Pg) exposure for 2 h. **b** The quantitative data of (**a**). **c** Confocal images of p65 expression in the HUVECs treated with Pg and/or NF-κB inhibitor BAY 11-7082 (BAY, 1 μmol·L^−1^) for 2 h. **d** Western blot analysis of p-p65, p65, and IκBα protein levels in the HUVECs treated with Pg and/or BAY (0.1, 0.5, 1 μmol·L^−1^) for 2 h. **e** The quantitative data of (**d**). **f** EdU staining of the HUVECs treated with Pg and/or BAY (1 μmol·L^−1^) for 48 h. Nuclei were stained with DAPI (blue). **g** The quantitative data of (**f**). **h** Transwell assays were used to test the migration of HUVECs treated with Pg and/or BAY (1 μmol·L^−1^) for 48 h. **i** The quantitative data of (**h**). **j** Apoptosis was evaluated by flow cytometry of the HUVECs treated with Pg and/or BAY (1 μmol·L^−1^) for 24 h. **k** The quantitative data of (**j**). Data were analysed using a two-tailed unpaired *t*-test (**b**) or one-way ANOVA (**e**, **g**, **i**, **k**) and are presented as the means ± SD. *P < 0.05; **P < 0.01; ***P < 0.001 compared to the control group. ns = not significant. ^#^*P* < 0.05; ^##^*P* < 0.01; ^###^*P* < 0.001 compared to the Pg group

Considering that TLRs regulate cell function mostly by triggering many other downstream signalling pathways^33^ and NF-κB is the common pathway that is also tightly associated with cardiovascular diseases,^34^ we proposed to further explore the link between NF-κB signalling and *P. gingivalis*-induced endothelial damage. BAY 11-7082, an NF-κB inhibitor, was employed. As shown in Fig. 5a, b, NF-κB signalling was activated upon *P. gingivalis* exposure. BAY 11-7082 significantly inhibited this pathway (Fig. 5c–e). Notably, we observed that BAY 11-7082 recovered half of the proliferative ability of HUVECs reduced by *P. gingivalis* (Fig. 5f, g) and attenuated approximately 60% of the migration and apoptosis of HUVECs induced by *P. gingivalis* (Fig. 5h–k). These results indicated that blockade of NF-κB signalling rescued the effects of *P. gingivalis* infection in HUVECs by at least half, suggesting that NF-κB signalling plays a critical role in the progression of *P. gingivalis*-destroyed endothelial homeostasis.

## Discussion

The vascular endothelium is a single cell layer that lines the entire circulatory system and is viewed as the first line of defence against cardiovascular disease. This structure is an interface between the bloodstream and the vessel wall. The endothelium can respond to several types of stimuli and regulate platelet function, inflammatory responses, and vascular smooth muscle cell growth and migration, in addition to modulating vascular tone by synthesizing and releasing vasoactive substances.^26^ The integrity of the endothelium plays a key role in maintaining its physiological function. Endothelial injury is believed to be of primary importance in the pathogenesis of vascular disease. When suffering prolonged and/or repeated exposure, endothelial cell protective activation is exhausted, thus leading to endothelial dysfunction, detachment and fragmentation into microparticles; consequently, the integrity is disrupted. The stimuli include high levels of lipoprotein,^35^ proinflammatory cytokines such as TNFα and IL-6 (ref. ^36^) and foreign pathogen infections such as viruses^37^ in the bloodstream. In our study, we found that a periodontal pathogen, *P. gingivalis*, is also such a stimulus. After exposure to *P. gingivalis*, we observed the loss of endothelial integrity in vivo.

The interaction between *P. gingivalis* and endothelial integrity has been studied for several years. It is well established that *P. gingivalis* has a direct route to the circulatory system by transient bacteraemia (the presence of bacteria in the blood) and that these events occur commonly and frequently.^38,39^ It has also been reported that *P. gingivalis* is highly invasive in several cell types, including endothelial cells.^40,41^ Studies have shown that *P. gingivalis* can adhere to and invade endothelial cells and stimulate the expression of proinflammatory cytokines, such as IL-6, IL-8 and MCP-1, and induce enhanced endothelial cell surface expression of VCAM-1, ICAM-1 and E-selectin^27^ which increases mononuclear cell adhesion and promotes vascular inflammation. *P. gingivalis* can also induce iNOS, inhibit eNOS expression and regulate the release of NO without any additional stimulant in endothelial cells,^28^ contributing to nitrative stress and endothelial function impairment. These studies all indicate that *P. gingivalis* can damage endothelial integrity, but they only focus on functional or biosynthetic aspects. The structural change of endothelial cells by *P. gingivalis* require further studies. Our data demonstrated that infection with *P. gingivalis* leads to increased apoptosis and EndMT of endothelial cells, damaging endothelial integrity. However, endothelial integrity depends not only on the extent of injury but also on the appropriate availability of functional endothelial cells. Notably, we detected a reduced proliferative ability of *P. gingivalis*-stimulated endothelial cells. These findings demonstrate that *P. gingivalis* not only impairs endothelial integrity but also inhibits its repair ability.

TLRs are a group of pathogen-associated molecular pattern (PAMP) recognition receptors that play an important role in innate immune signalling in response to microbial infection. It has been suggested that TLR signalling may be activated and that TLR expression may be altered in cardiovascular disease.^42,43^ Specific TLRs have demonstrated functional pattern recognition of peptidoglycan and bacterial lipopeptides (TLR2), double-stranded RNA (TLR3), lipopolysaccharide (LPS; TLR4), flagellin (TLR5), and unmethylated CpG DNA motifs (TLR9).^44^ Among these TLRs, TLR2 and TLR4 are the most common TLRs in the host response to *P. gingivalis* lipopolysaccharides^45^ and fimbriae.^46^ Previous studies have demonstrated that *P. gingivalis* can colocalize with TLR2 and manipulate TLR2 downstream signalling to uncouple bacterial clearance from inflammation and promote dysbiosis.^32^ *P. gingivalis* also invades HAECs by TLR4.^47^ Consistent with these studies, we also found that TLR signalling was activated after *P. gingivalis* was added, and the effects of *P. gingivalis* on HUVECs were reversed almost completely when TLR signalling was inhibited. TLRs frequently coexist with NF-κB, which induces the expression of TLRs and regulates the downstream signalling of TLRs on ligand engagement.^48–50^ In the present study, we detected the activation of NF-κB signalling in *P. gingivalis*-infected HUVECs. After addition of the inhibitor BAY 11-7082, the effects of *P. gingivalis* were attenuated by ~60%, suggesting that the remaining effects (~40%) might come from other downstream mechanisms of TLRs, which need further research.

Our data confirmed the critical role of TLR-NF-κB signalling in the progression of *P. gingivalis*-destroyed endothelial homeostasis and suggest that specific inhibition of TLR-NF-κB activation by *P. gingivalis* concomitantly reduces the risk of infection-accelerated cardiovascular disease. This strategy may slow the progression of cardiovascular disease by regulating possible therapeutic targets.

Cardiovascular disease is a multifactorial disease, and the reason why it can cause endothelial dysfunction is also complex. Here, by using *P. gingivalis* via the bloodstream for assessment of destroyed endothelium, we demonstrated an experimental link between *P. gingivalis* and endothelial homeostasis, which also suggests the negative impact of periodontal pathogens on cardiovascular disease. In addition, studies have shown that periodontal treatment can result in a reduction in systemic inflammation and endothelial dysfunction,^51^ which further confirms the importance of periodontal intervention in preventing or treating cardiovascular disease. Although there is no overwhelming evidence that periodontal therapy can prevent cardiovascular disease or modify its outcomes, according to the results of our study, it is still necessary for oral clinicians to remind patients to pay attention to periodontal health.

## Materials and methods

### Bacterial culture

*P. gingivalis* W83 was kindly provided by the West China College of Stomatology, Sichuan University, China. *P. gingivalis* was grown on anaerobic blood agar plates in a chamber with 85% N_2_, 5% H_2_, and 10% CO_2_ at 37 °C for 5–7 days. It was then inoculated into a liquid broth of brain heart infusion (BHI) supplemented with hemin (5 µg·mL^−1^) and vitamin K (5 µg·mL^−1^) for over 24 h in the same chamber.

### Mice

Ten male Balb/c mice of 6 weeks of age were used in this study. Mice were randomly divided into two groups (*n* = 5 per group): (1) control group, mice administered 0.1 mL of PBS via tail-vein injection once a week; (2) Pg group, mice with *P. gingivalis* (10^7^ CFU in 0.1 mL PBS) administered via tail-vein injection once a week. All mice were kept in a standard environment with controlled temperature and humidity under specific-pathogen-free conditions within individually ventilated cages with food and water ad libitum. After 6 weeks, the mice were euthanized at the same time, and the tissues were collected. The thoracic abdominal aorta and brachiocephalic artery were fixed in 4% paraformaldehyde (PFA) for further detection. There were no observable behavioural changes in the mice injected with *P. gingivalis* or PBS throughout the study. All animal experiments were carried out with the consent of the Institutional Animal Care and Use Committee of Tongji Medical College (Wuhan, China).

### Immunostaining and immunohistochemistry

For the morphological detection of aortas, haematoxylin-eosin staining (H&E) was implemented following routine procedures. Briefly, after deparaffinization and rehydration, sections were stained with haematoxylin solution for 5 min, followed by incubation with acidic ethanol and rinsing in distilled water. The sections were stained with eosin solution for 3 min followed by dehydration and clearing. Morphological features of the indicated specimens were observed under optical microscopy.

For the detection of apoptosis, a TUNEL assay was performed. Tissue sections of brachiocephalic arteries from the sacrificed mice were incubated in xylene and rehydrated in decreasing concentrations of alcohol, after which the sections were treated with 3% EDTA at a pH of 7.2 for 1 h. Endogenous peroxidase activity was inactivated by 3% H_2_O_2_. The TUNEL assay was then performed using an in situ Apoptosis Detection Kit (TaKaRa), following instructions from the manufacturer.

For immunohistochemistry analysis, mouse paraffin-embedded aortic tissue sections were incubated with primary antibody (CD31 (Abcam)) overnight at 4 °C. Then, the sections were washed and incubated with secondary antibody conjugated with horseradish peroxidase for 1 h at room temperature. The expression of the various molecules was visualized using DAB substrate, which reacts with horseradish peroxidase to yield a brown coloured product on the tissue sections.

### Cell culture and reagents

Human umbilical vein endothelial cells (HUVECs; ScienCell) and human aortic endothelial cells (HAECs; ScienCell) were cultured in endothelial cell medium (ScienCell) supplemented with 5% foetal bovine serum and 1% endothelial cell growth factors. Cells were maintained at 37 °C in a humidified atmosphere of 5% CO_2_ in air. The passage of cells was usually 4 to 6. OxPAPC (Invitrogen) and BAY11–7082 (MedChemExpress) were used as inhibitors.

### Quantitative reverse transcriptase-polymerase chain reaction (qRT-PCR)

Total RNA from HUVECs was isolated by TRIzol reagent (TaKaRa). Reverse transcription was performed using a Superscript II-reverse transcriptase kit (Vazyme). qRT-PCR was performed using Subsequent Fast SYBR Green qPCR on Step One Plus real-time PCR systems (Applied Biosystems) according to the manufacturer’s instructions. The sequences of the primers used for qRT-PCR are described in Table 1. Quantification of target gene expression was calculated relative to the reference glyceraldehyde 3-phosphate dehydrogenase (GAPDH) gene. The mean minimal cycle threshold values (Ct) were calculated from triplicate reactions.

**Table 1:** Primers for quantitative reverse transcription-polymerase chain reaction (qRT-PCR)

Genes	Forward primer 5′–3′	Reverse primer 5′–3′
ACTA2	TGAGCGTGGCTATTCCTTCGTT	GCCCATCAGGCAACTCGTAACT
CD31	AACAGTGTTGACATGAAGAGCC	TGTAAAACAGCACGTCATCCTT
FSP-1	GCTCAACAAGTCAGAACTAAAGGAG	GCAGCTTCATCTGTCCTTTTC
VE-cad	TTGGAACCAGATGCACATTGAT	TCTTGCGACTCACGCTTGAC
GAPDH	GGAGCGAGATCCCTCCAAAAT	GGCTGTTGTCATACTTCTCATGG

### Western blot analysis

Total cellular proteins were extracted using RIPA buffer with fresh protease inhibitors (InvivoGen) and phosphatase inhibitors (InvivoGen). The concentration of proteins was quantified using the BCA kit (Beyotime). The proteins were separated by electrophoresis on a 10% SDS-PAGE gel and then transferred onto a PVDF membrane. After blocking for 1 h with skim milk, the membranes were incubated with primary antibodies against ACTA2 (Proteintech), BAX (Proteintech), BCL2 (Proteintech), Caspase 3 (Cell Signaling Technology), CD31 (Abcam), FSP-1 (Proteintech), VE-cadherin (Proteintech), Vimentin (Proteintech) and GAPDH (Proteintech) overnight at 4 °C. Then, the cells were incubated with secondary antibodies for more than 1 h. The protein-antibody complexes were then visualized using the enhanced chemiluminescence detection system. Quantification of the protein expression level was calculated using ImageJ software.

### Apoptosis analysis

In HUVECs, apoptosis induced by *P. gingivalis* was detected quantitatively with an Annexin-V-fluorescein isothiocyanate (FITC) apoptosis detection kit (InvivoGen). Briefly, cells were collected and washed with cold PBS. Then, they were incubated and stained with FITC-Annexin V at room temperature in the dark for 15 min followed by PI addition. The cells were analysed by a BD FACS Canto ІІ Flow Cytometer. Data are expressed as the mean of total apoptosis, representing the sum of early and late apoptosis.

### Morphological evaluation

HUVECs were seeded into 6-well plates at a proper density, after which infection of *P. gingivalis* was carried out. Incubation of *P. gingivalis* with HUVECs was performed for 48 h. Cells were washed with PBS three times and fixed with 4% PFA. Morphological changes were then analysed using a microscope.

### Immunofluorescence staining

Cells were fixed with 4% paraformaldehyde for 10 min, washed three times with PBS, and treated with 0.1% Triton X-100 for 10 min. Next, primary antibodies against CD31 (Abcam), ACTA2 (Proteintech) and Ki67 (Abcam) were applied and incubated overnight at 4 °C in a humidified chamber. After extensive rinsing with PBS, the coverslips were incubated with secondary antibody (488-labelled goat anti-rabbit antibody, Proteintech) for 2 h at room temperature. After three washes with PBS, cell nuclei were counterstained with DAPI. Immunofluorescence photographs were taken by a fluorescent confocal microscope (Nikon A1-Si) with NIS software for acquisition.

### Cell migration assay

The migration assay was performed using a modified Boyden chamber apparatus (Transwell apparatus, 8.0-μm pore size, Corning). An appropriate number of HUVECs was seeded into the upper chamber with serum-free culture medium, and complete medium was added to the lower chambers of the inserts in 24-well plates. After infection with *P. gingivalis* at 37 °C for 48 h, HUVECs were fixed in 4% paraformaldehyde and stained with 1% crystal violet. Nonmigrated HUVECs were removed from the upper face of the Transwell membrane with a cotton swab. The migrated cells were manually calculated in five random microscopic fields using a microscope.

### Cell viability

Cell Counting Kit-8 (CCK-8) (Beyotime) was used to measure cell viability. HUVECs were seeded in 96-well plates at a density of 2 × 10^3^ cells per well. Cells were exposed to *P. gingivalis* with or without inhibitor pretreatment. After the indicated times, the cells were incubated with 10 μl CCK-8 solution at 37 °C for 1 h following the manufacturer’s instructions. Optical density (OD) values at 450 nm were obtained using a microplate reader.

### EdU staining

HUVECs and HAECs were seeded in 12-well plates at a degree of fusion of 50% cells per well. Cells were exposed to *P. gingivalis* with or without inhibitor pretreatment. After 48 h, EdU staining was chosen for direct observation of cell proliferation. Experimental procedures were performed according to the manufacturer’s protocols (Beyotime). Briefly, click working solution was mixed following the instructions. Cell samples were then incubated with working solution for 30 min at room temperature without light. Hoechst was selected for nuclear staining. Washing buffer was used to clean the residual solution. Immunofluorescence images were obtained by fluorescence microscopy. The proportions of cells showing EdU labelling were quantified using the ImageJ programme.

### Statistical analysis

Statistical significance was assessed using Student’s *t*-test or analysis of variance (ANOVA). The results are presented as the mean ± standard deviation (SD). A *p* value < 0.05 was considered statistically significant, and all statistical analyses were performed with GraphPad Prism 7.0 software.
